# An informatics guided classification of miscible and immiscible binary alloy systems

**DOI:** 10.1038/s41598-017-09704-1

**Published:** 2017-08-29

**Authors:** R. F. Zhang, X. F. Kong, H. T. Wang, S. H. Zhang, D. Legut, S. H. Sheng, S. Srinivasan, K. Rajan, T. C. Germann

**Affiliations:** 1 School of Materials Science and Engineering, and International Research Institute for Multidisciplinary Science, Beihang University, Beijing, 100191 P. R. China; 2 CAS Key Laboratory of Nuclear Materials and Safety Assessment, Institute of Metal Research, Chinese Academy of Sciences, Shenyang, 110016 P.R. China; 3 IT4Innovations Center & Nanotechnology Centre, VSB-Technical University of Ostrava, CZ-70833 Ostrava, Czech Republic; 4 Plant Sciences Institute, Iowa State University, 2031 Roy J. Carver Co-Lab, Ames, IA 50011 USA; 5 Department of Materials Design and Innovation, University at Buffalo-State University of New York, 311 Bell Hall, Buffalo, NY 14260 USA; 6 Theoretical Division, Los Alamos National Laboratory, Los Alamos, NM 87545 USA

## Abstract

The classification of miscible and immiscible systems of binary alloys plays a critical role in the design of multicomponent alloys. By mining data from hundreds of experimental phase diagrams, and thousands of thermodynamic data sets from experiments and high-throughput first-principles (HTFP) calculations, we have obtained a comprehensive classification of alloying behavior for 813 binary alloy systems consisting of transition and lanthanide metals. Among several physics-based descriptors, the slightly modified Pettifor chemical scale provides a unique two-dimensional map that divides the miscible and immiscible systems into distinctly clustered regions. Based on an artificial neural network algorithm and elemental similarity, the miscibility of the unknown systems is further predicted and a complete miscibility map is thus obtained. Impressively, the classification by the miscibility map yields a robust validation on the capability of the well-known Miedema’s theory (95% agreement) and shows good agreement with the HTFP method (90% agreement). Our results demonstrate that a state-of-the-art physics-guided data mining can provide an efficient pathway for knowledge discovery in the next generation of materials design.

## Introduction

The purpose of the Materials Genome Initiative (MGI) is to accelerate the discovery of novel materials by means of modern computational techniques and data mining methods^1–3^. Successful examples reported so far include solar water splitters, solar photovoltaics, topological insulators, scintillators, clean energy materials, piezoelectrics, thermoelectrics, catalysts, hydrogen storage materials, and Li-ion batteries^2, 3^. With the emergence of MGI, an exciting trend has recently appeared, utilizing collaborative informatics technology, e.g. data collection and data-mining, to extract meaningful information and patterns from massive data for the fast design and development of novel materials^4^. The core task is to efficiently leverage various open-source data with state-of-the-art data-mining algorithms. In the fields of physical metallurgy, vast new data have been recently collected and reported for the mixing properties of binary alloy systems. This provides a unique opportunity to construct a visual miscibility map for binary alloy systems by mining these large numbers of data sources.

The miscibility of a binary alloy system, which indicates the tendency of two elements to form either a homogeneous solid solution or an intermetallic compound, plays an important role in the development of high-performance metallic materials such as diffusion barrier materials, bulk metallic glasses, damage-resistant nanomaterials, microelectronics, or superalloys^5, 6^. From a thermodynamical point of view, an immiscible system corresponds to a positive (i.e., endothermic) formation enthalpy^7–9^ (e.g., Cu-Nb systems^10, 11^). At the other extreme, a binary alloy system may readily form intermetallic compounds, or a homogeneous or clustered solid solution, typically with a solubility of about 10%^5, 12–14^. For such miscible cases, the alloying process is exothermic, with a negative formation enthalpy (e.g., Cu-Ti system)^15^. Several heuristic rules have been proposed to qualitatively predict the alloying behavior or reactivity of two metals, of which the most important ones are: the electronegativity rule^16^, the Hume-Rothery rule^17^ and the Darken and Gurry maps^18, 19^. A very successful semi-quantitative theory has been proposed by Miedema *et al*.^12^ in the late 1970’s to distinguish the negative (miscible) and positive (immiscible) systems. This model makes it possible to predict the formation enthalpies of binary alloy systems in a semi-quantitative manner^20^. However, the model is sensitive to the choice of its parameters, which are determined empirically. The most critical problems with Miedema’s model are: a) it needs more reliable experimental data to validate, improve and extend it^12, 13, 21–23^; b) as will be shown later, a large scatter of the results occurs when the formation enthalpy of the system is close to zero, i.e., for weakly alloying systems; and c) several outliers need be sorted out before it can be reliably used to guide the development of novel alloys.

The validation of such empirical and semi-quantitative rules depends generally on the availability of reliable and sufficient thermodynamic data. The recent progress in the development of the experimental techniques, thermodynamic analysis and modern first-principles methods provides an opportunity to improve the database of alloying behavior. For example, Kleppa *et al*.^24–35^ published experimental values of formation enthalpies with high accuracy, leading to updates in the Landolt-Boernstein database^7^, the handbook of binary alloy phase diagrams compiled by Okamoto^9^, and the alloy phase diagram database organized by ASM community^8^. In addition, modern high-throughput first-principles (HTFP) calculations^2, 36–40^ provide a unique way to check and compare the database from different sources. All of these data sources provide not only a pathway for a global data mining of alloying behavior of binary alloy systems, but an opportunity to validate the widely used Miedema theory and compare with HTFP method^21–23^.

In this article we first present a global collection and survey of the alloying behavior of binary systems based on the most recent phase diagrams, thermodynamic data and HTFP calculations. Second, we propose a data mining scheme to classify miscible and immiscible systems based on a general consideration of thermodynamic rules. Note that such data mining is not a trivial task; one may obtain a misleading conclusion if the data interpretation is not accompanied by a proper statistical analysis. It should be also noted that the requirement of massive data provides a foundation of the reliability of the present scheme for data mining, which however limits the present method to be used when sufficient data are unavailable for statistical analysis. Third, a series of two-dimensional miscibility maps are constructed based on several physically relevant descriptors to distinguish the immiscible systems from miscible ones by pattern recognition. Fourth, the neural network algorithm and elemental similarity are used to predict the alloying behavior of unknown systems, thus providing a complete miscibility map. Finally, a thorough validation of the classical Miedema theory and a comprehensive comparison with HTFP method is performed by statistical analysis.

## Results

### Classification of miscible and immiscible alloy systems

In all, 44 metallic elements comprise the 27 transition metals (TM), including Zn, Cd and Hg, and 17 lanthanide (rare earth (RE)) metals, including Sc and Y. Because of the scarcity of experimental and HTFP data for binary alloy systems consisting of binary lanthanide metals, i.e. RE-RE, we shall not discuss this group except for three binary systems of Sc-Y, Sc-La and La-Y. Consequently, we investigate a total of ${C}_{27}^{2}$ = 351 + 459 + 3 = 813 binary alloy systems in the present study. The classification of different solid solutions (with positive and negative formation enthalpy) is not an easy task since there is no direct unified experimental data to distinguish them. However, the formation enthalpy provides an energetic criterion for this purpose. After a global survey, we found 35 binary alloy systems where a disagreement appeared between phase diagram analysis and HTFP calculations. These are listed in Table 1 as “outliers”. As regards this group, our final choice of miscibility is based on an evaluation of the available experimental information, the similarity of chemical elements, and a global justification by comparison of different data sources. Remarks are added in the last column: SSL: low solid solubility; SSH: high solid solubility; COM: formation of compounds; COM1: only one compound reported; IMM: immiscibility gap exists.

**Table 1:** The binary alloy systems disagreed between phase diagram information and high throughput first principles (HTFP) calculations.

Alloy systems	Alloying behavior			Remarks^*^
	HTFP	Phase diagram	Present choice	
Ag-Hg	+	−	−	COM
Au-Cr	+	−	+	COM1
Au-Hg	+	−	−	COM
Cd-Cu	+	−	−	COM
Cd-Nb	+	−	+	COM
Co-Cr	+	−	−	COM
Co-Ir	+	−	−	SSH
Co-Os	+	−	−	SSH
Co-Rh	+	−	−	SSH
Co-Ru	+	−	−	SSH
Cr-Fe	+	−	−	COM1
Cr-Mn	+	−	−	COM
Cr-Re	+	−	−	SSH
Cr-Ru	+	−	−	SSH
Cr-Tc	+	−	−	COM
Cr-Zn	+	−	−	COM
Cu-Hg	+	−	−	COM1
Cu-Nb	−	+	+	IMM
Fe-Os	+	−	+	SSH
Fe-Ru	+	−	+	SSH
Hf-V	+	−	−	COM
Hg-Mn	+	−	−	COM
Hg-Ni	+	−	−	COM
Hg-Zn	+	−	−	COM
Mn-W	−	+	−	IMM
Mn-Y	+	−	−	COM
Mo-Sc	−	+	+	SSL
Mo-Tc	+	−	−	COM
Ni-Re	−	+	−	SSL
Os-Pt	+	−	+	SSH
Re-W	+	−	−	COM
Sc-Y	+	−	+	SSH
Tc-W	+	−	−	COM
Ti-Zr	+	−	+	SSH
V-Zr	+	−	−	COM1

The present choice is based on the estimation on the reliability of available information of phase diagram and thermodynamic data and elemental similarity. Plus symbol represents the immiscible systems, and minus symbol indicates the miscible or compound-formation systems.

*Note: SSL: low solid solubility; SSH: high solid solubility; COM: formation of multiple compounds; COM1: only one compound reported; IMM: immiscibility gap.

Special binary systems deserve particular attention. For the Ag-Hg, Au-Hg, Co-Cr, Cr-Mn, Cr-Tc, Cr-Zn, Hg-Mn, Hg-Ni, Hg-Zn, Mn-Y, Mo-Tc, Re-W, and Tc-W systems, the formation of stable intermetallic compounds have been reported in the published phase diagrams. Thus, we believe that these systems should be identified as miscible systems with a negative formation enthalpy, although the HTFP calculations provide opposite results. For Ag-Mn, Au-Cr, Fe-Os, Fe-Ru, Mn-W, Os-Pt, and Ta-Ti, the HTFP calculations are accepted because there is no information on phase diagrams or thermodynamic data available. For other binary alloy systems listed in Table 1, our judgment is based on the elemental similarity, as will be discussed later.

### Selection of physical descriptors

In order to enable pattern recognition, several physical descriptors, i.e. semi-empirical parameters and scales, are considered for a systematic description (or prediction) of the alloying behavior of the different metals. The physical descriptors are based on the similarity of atomic, physical and chemical properties of the elements, such as the atomic number (AN), Pauling electronegativity (PEN)^16, 41, 42^, Teatum metallic radii of elements with coordinate number CN12 (Rij)^43–45^, Martynov-Batsanov electronegativity (MBEN)^35, 46^, Zunger’s pseudopotential radii sum (Rsp)^35, 47^, Miedema’s work function (Phi)^5, 14^, Miedema’s electron density at the Wigner-Seitz cell boundary (Nws)^5, 14^, Miedema’s molar volume (Vm)^5, 14^, and Mendeleev number or Pettifor chemical scale (Pcs)^48^. The choice of these parameters or scales is based on the success of these parameters in the description of different alloying behaviors.

The first choice is the atomic number, which uniquely identifies a chemical element. The second criterion is based on the consideration of the diagrams developed by Darken and Gurry for solid solution predictions^19^. The two coordinates represent the Pauling electronegativity and the atomic size of the elements. Electronegativity is a chemical property that describes the tendency of an atom or a functional group to attract electrons (or electron density). Pauling originally proposed the concept of electronegativity as an explanation of the fact that the covalent bond between two different atoms (A-B) is stronger than that would be expected by taking the average of the strengths of the A-A and B-B bonds. Allred^16, 41, 42^ updated Pauling’s original values by taking into account the available thermodynamic data. These “revised Pauling” values of the electronegativity are most often used.

The atomic radius of a chemical element is usually the mean or typical distance from the nucleus to the boundary of the surrounding cloud of electrons. Since the boundary is not well-defined, there are various non-equivalent definitions of atomic radii. Four widely used definitions are the Van der Waals radius, ionic radius, covalent radius and metallic radius. Among these definitions, the metallic radius proposed by Teatum^43–45^ is probably the most useful and widely accepted for metallic alloys, and will be adopted in the present work.

The third group of parameters come from the work by Villars, who analyzed the behavior of alloy systems on the properties of the component elements^35^. He used a systematic elimination procedure to find atomic properties which could be used to distinguish the crystal structures of intermetallic compounds. 182 sets of tabulated physical properties and calculated atomic properties were considered. The best separation was obtained using three dimensional maps with the following variables as coordinates: (1) the mean number of valence electrons of the two elements, (2) the electronegativity difference proposed by Martynov-Batsanov in 1980^35, 46^, and (3) the difference of Zunger’s pseudopotential radii sum^35, 47^. In view of the success of Villars’ structure map, the two critical parameters of Martynov-Batsanov electronegativity and Zunger’s pseudopotential radii sum are adopted in the present study.

The fourth group of parameters is derived from the famous Miedema’s model because of its success in the description of the energy effects during alloying. Positive (immiscible) and negative (miscible) systems can be separated by two parameters of the constituent chemical elements: the chemical potential for electronic charge (electronegativity), Phi and the electron density at the boundary of the Wigner-Seitz atomic cell, Nws. Miedema’s model became very popular due to the scarcity of experimental data, and the model provided a semi-quantitative evaluation. By assigning the two parameters as two coordinates it was possible to build a map in which a clear separation was observed between all binary alloys with positive heats of formation and those with negative values^5, 12–14^. Additional justification will be provided later, in section 3.5. A quantum-mechanical interpretation of Miedema’s parameters has been proposed by Chelikowsky and Phillips^49^.

The last criterion is based on Pettifor’s work on the chemical scale (or Mendleev number, M)^48^. This has been set up by ordering the elements along a single axis so that the Mendeleev-type features of the periodic table are preserved. The Mendeleev numbers start with the least electronegative elements and end with the most electronegative ones. By using them, an excellent separation of similar structures is achieved for numerous A_m_B_n_ phases with a given stoichiometry within M_A_-M_B_ maps. Although the chemical scale is entirely phenomenological and has no a priori significance other than that it orders the elements relative to each other, Pettifor has shown that the corresponding two-dimensional structure maps achieve excellent structural separation. It will be shown in the following sections that the progressive order of chemical scale provides an excellent pattern clustering in the miscibility map of binary alloy systems.

Table S1 (in the Supplemental Materials) lists the adopted values of Pauling electronegativity (Pen)^16, 41, 42^, Teatum metallic radii of elements with coordinate number CN12 (Rij)^43–45^, Martynov-Batsanov electronegativity (Men)^35, 46^, Zunger’s pseudopotential radii sum (Rsp)^35, 47^, Miedema’s work function (Phi)^5, 14^, Miedema’s electron density at the Wigner-Seitz cell boundary (Nws)^5, 14^, Miedema’s molar volume (Vm)^5, 14^, the Mendeleev number or the Pettifor chemical scale (Pcs)^48^, and atomic number (AN) for ordering the 44 elements (27 transition metals and 17 Lanthanides). We refer the reader to the original publications on these parameters for the detailed physical meaning and their derivations.

### The miscibility map sorted by different descriptors

We use the descriptors (“parameters”) defined in the previous section to analyze the pattern as it appears in the two-dimensional miscibility maps. Figure S1 (in the Supplemental Materials) shows the miscibility map ordered by Atomic number, Pauling electronegativity, Teatum metallic atomic radii, Martynov-Batsanov electronegativity, and Zunger’s pseudo radii sum, respectively. These physical descriptors belong to the first three groups of variables identified in the preceding section. It can be seen that the atomic number, Pauling electronegativity, Teatum metallic atomic radii, Martynov-Batsanov electronegativity, and Zunger’s pseudo radii sum can distinguish (“cluster”) the immiscible systems distinctly. This indicates that these parameters provide elemental similarity and the underlying physics. However, the clustering patterns ordered by Pauling electronegativity, Teatum metallic atomic radii, Martynov-Batsanov electronegativity, and Zunger’s pseudo radii, do not provide any better feature identification than that by atomic number. Interestingly, the descriptors belonging to the fourth and fifth group of variables are found to divide the immiscible systems within clusters much better than those in the former three criteria. Figure S2 (in the Supplemental Materials) shows the clustering feature of immiscible alloy systems ordered by Miedema’s electronegativity, Miedema’s electron density, Miedema’s molar volume, and especially by Mendeleev number or the Pettifor chemical scale. Although Miedema’s two parameters were shown to be a great success for predicting the formation enthalpies of intermetallic compounds, no single one provides a better ordering sequence than those according to criteria 1, 2 and 3. We further see that the Pettifor chemical scale shows the best separation between the miscible and immiscible systems and the distinct clustering of immiscible systems.

**Figure 1:**
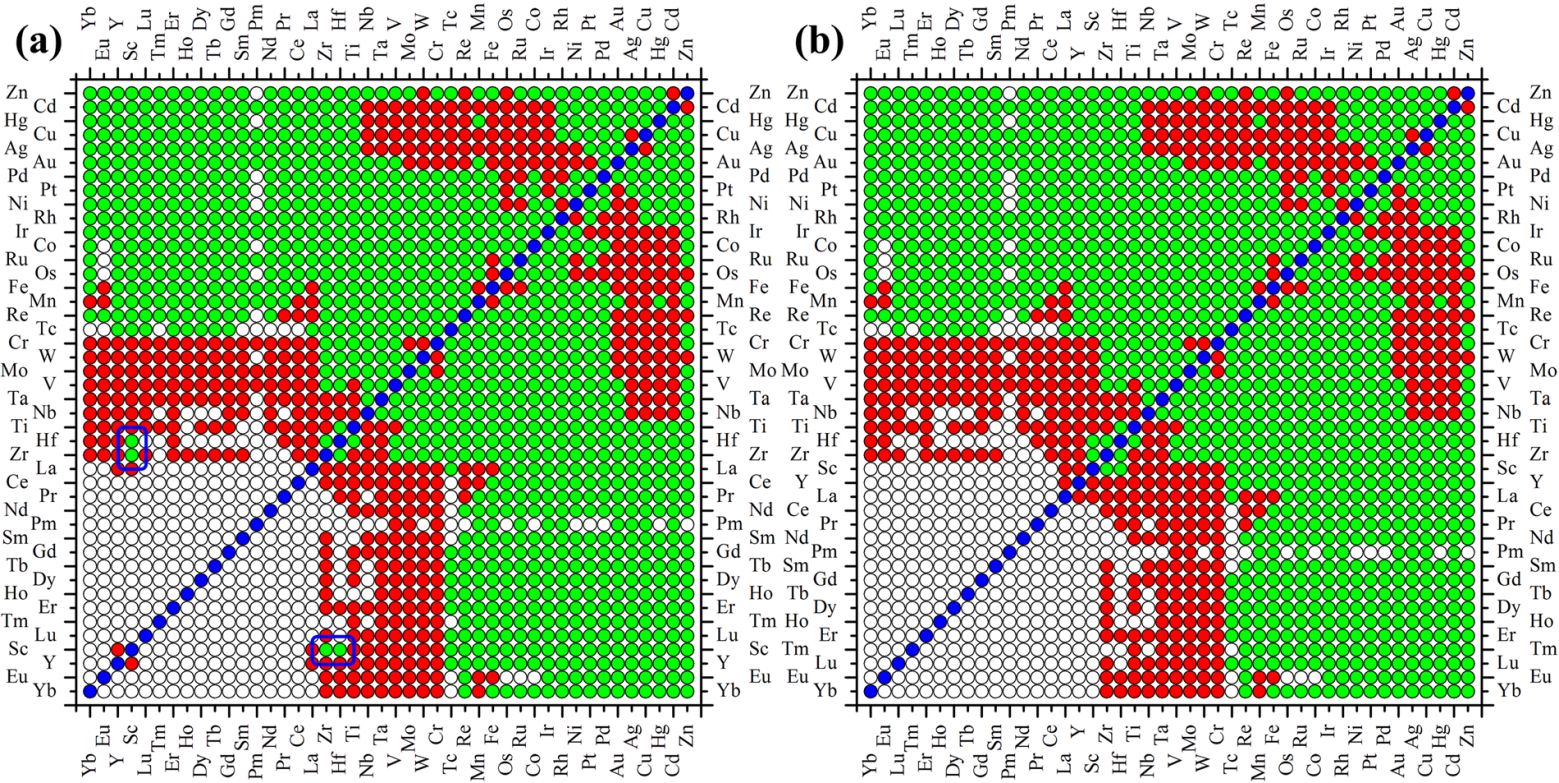
The incomplete miscibility maps of binary alloy systems ordered by (**a**) the original Pettifor chemical scale and (**b)** the slightly modified ordering the RE elements. The red and green symbols indicate immiscible and miscible systems, respectively. The white symbols represent the systems with the unavailable experimental information and are not considered in the present study, while the blue ones indicate the boundary of alloys consisting of dissimilar elements.

After an in-depth analysis of the pattern feature ordered by Mendeleev number (Pettifor chemical scale), we found that some outliers appear to break the continuous clustering feature for the lanthanide-based systems (see the two green points circled by blue squares in Fig. 1a). In order to avoid this breaking of continuity of the distribution of immiscible systems, we slightly modify the order of rare earth/lanthanide elements. Figure 1b shows the modified miscibility map ordered by the modified Mendeleev number or the Pettifor chemical scale with the new elemental ordering as: Yb < Eu < Lu < Tm < Er < Ho < Dy < Tb < Gd < Sm < Pm < Nd < Pr < Ce < La < Y < Sc < Zr < Hf < Ti < Nb < Ta < V < Mo < W < Cr < Tc < Re < Mn < Fe < Os < Ru < Co < Ir < Rh < Ni < Pt < Pd < Au < Ag < Cu < Hg < Cd < Zn. It is seen from Fig. 1b that the new elemental sequence clearly divides the immiscible systems into two major clustered regions. Such clustering can be understood via similarity and dissimilarity of the elements in physics and chemistry.

### Prediction of unknowns via artificial neural network algorithm

**Figure 2:**
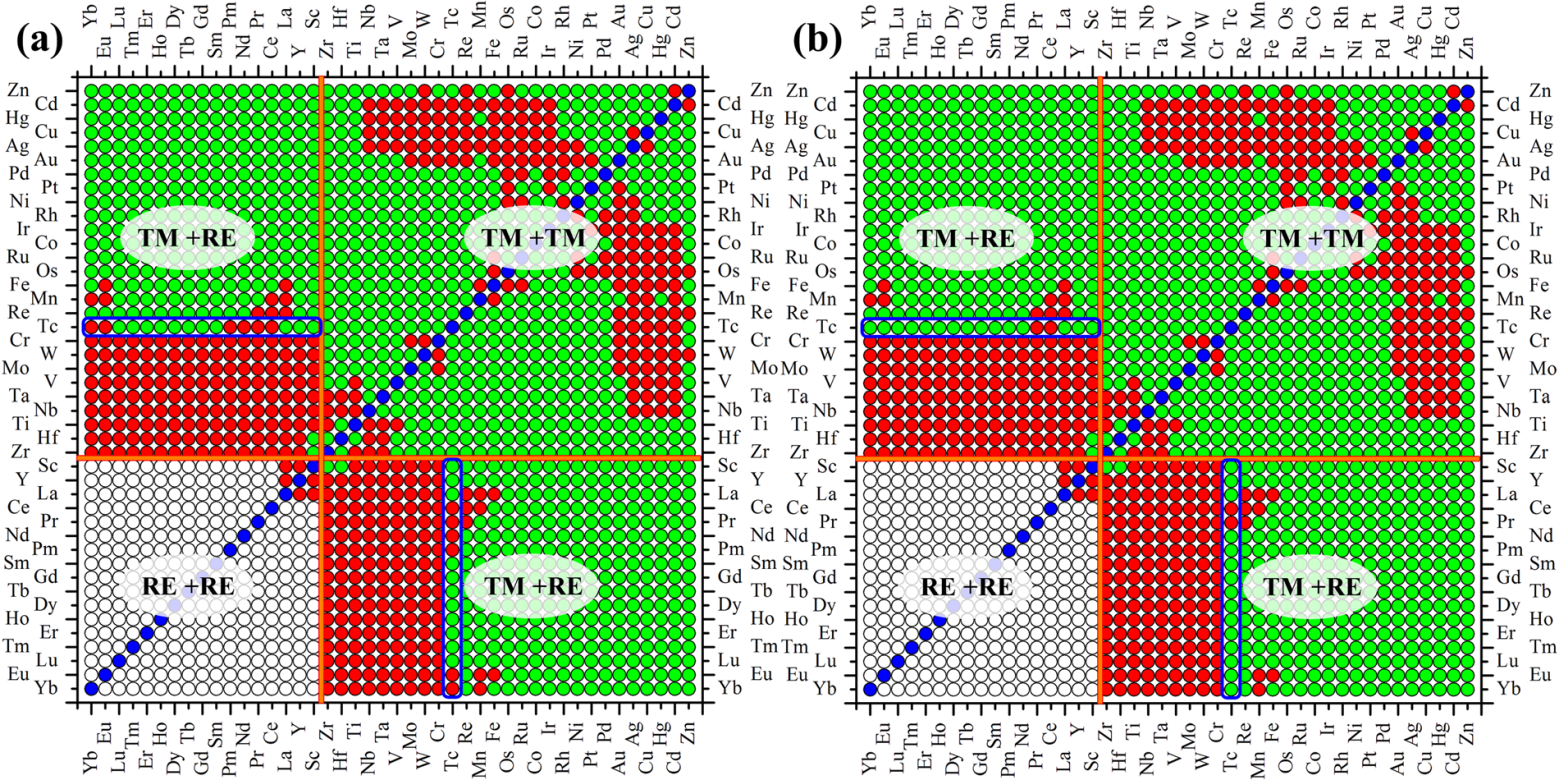
The complete miscibility map of binary alloy systems ordered by the slightly modified Pettifor chemical scale based on (**a**) the RBF-ANN analysis, (**b**) the RBF-ANN analysis plus elemental similarity between Re and Tc.

We now apply the incomplete miscibility maps (Fig. 1b) and the radial basis function artificial neural network (RBF-ANN) algorithm as described in Methods section to predict the unknown classification of binary alloy systems in order to obtain a complete miscibility map. Figure 2a shows the filled miscibility map of binary alloy systems ordered by the slightly modified Mendeleev number or the Pettifor chemical scale based on the RBF-ANN analysis. The three combinations are binary transition metal alloys (TM + TM), a combination of transition metals and rare earth metals (TM + RE), and a binary combinations of rare earth metals (RE + RE). Two immiscible regions are located at TM + RE, and TM-TM combinations as seen in Fig. 2a.

It is seen that the prediction is mostly in agreement with the empirical prediction based on simple elemental similarity, with a few outliers for Tc-based systems. The agreement between the elemental similarity and the RBF-ANN analysis can be summarized as follows:

1. For Pm-TM binary alloy systems: based on the similarity between Pm, Sm and Nd, one may see that the transition metals Zr < Hf < Ti < Nb < Ta < V < Mo < W < Cr are immiscible with Pm, whereas Pm is miscible with Tc < Re < Mn < Fe < Os < Ru < Co < Ir < Rh < Ni < Pt < Pd < Au < Ag < Cu < Hg < Cd < Zn.
2. For binary alloy systems between elements consisting of Zr < Hf < Ti < Nb < Ta < V < Mo < W < Cr and Yb < Eu < Lu < Tm < Er < Ho < Dy < Tb < Gd < Sm < Pm < Nd < Pr < Ce, the immiscibility is readily chosen for these classes of binary alloy systems based on the elemental similarity.
3. For Eu-(Co, Ru, Os) binary alloy systems: based on the elemental similarity between Rh(Ir) and Ru(Os), a miscible alloying behavior is an appropriate choice for the three Eu-(Co, Ru, Os) binary alloy systems.

**Figure 3:**
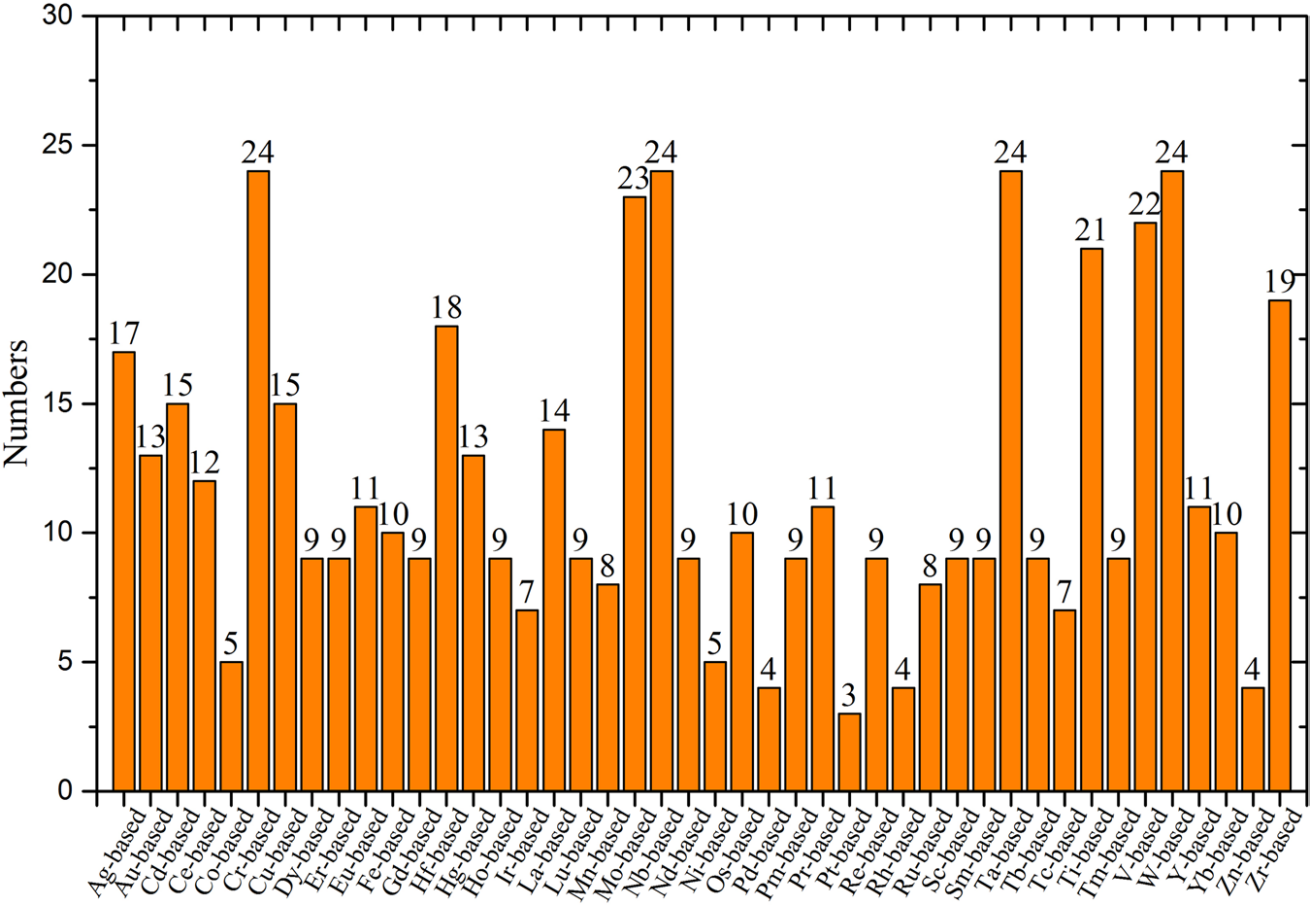
The numbers of immiscible binary alloy systems of different M-based binary systems (M is transition metals or rare earth metals). Significant feature is found that Cr, Nb, Ta, W, Mo -based systems possess the most number of immiscibility.

Because the binary Tc-based alloy systems are located at the boundary of miscible and immiscible systems, one needs to further justify the miscibility of the related outliers based on the elemental similarity. Although our RBF-ANN analysis suggests that Tc-Eu, Tc-Yb, Tc-Pm and Tc-Nd systems are mostly immiscible, the elemental similarity between Tc and Re give an opposite conclusion, i.e. these systems should be miscible (negative formation enthalpy). Based on the similarity of valence electrons between Re and Tc, we would classify the four systems as miscible ones, and accordingly, Fig. 2b gives the complete miscibility map. Table S2 (in the Supplemental Materials) lists all of the 262 immiscible binary alloy systems identified from the 813 candidates. Figure 3 shows the numbers of the immiscible binary alloys formed from different transition or rare earth metals M. It is seen that Cr, Nb, Ta, W, Mo -based binaries have the largest number of immiscible elements.

### Comparison with Miedema’s theory and high throughput first principles methods

**Figure 4:**
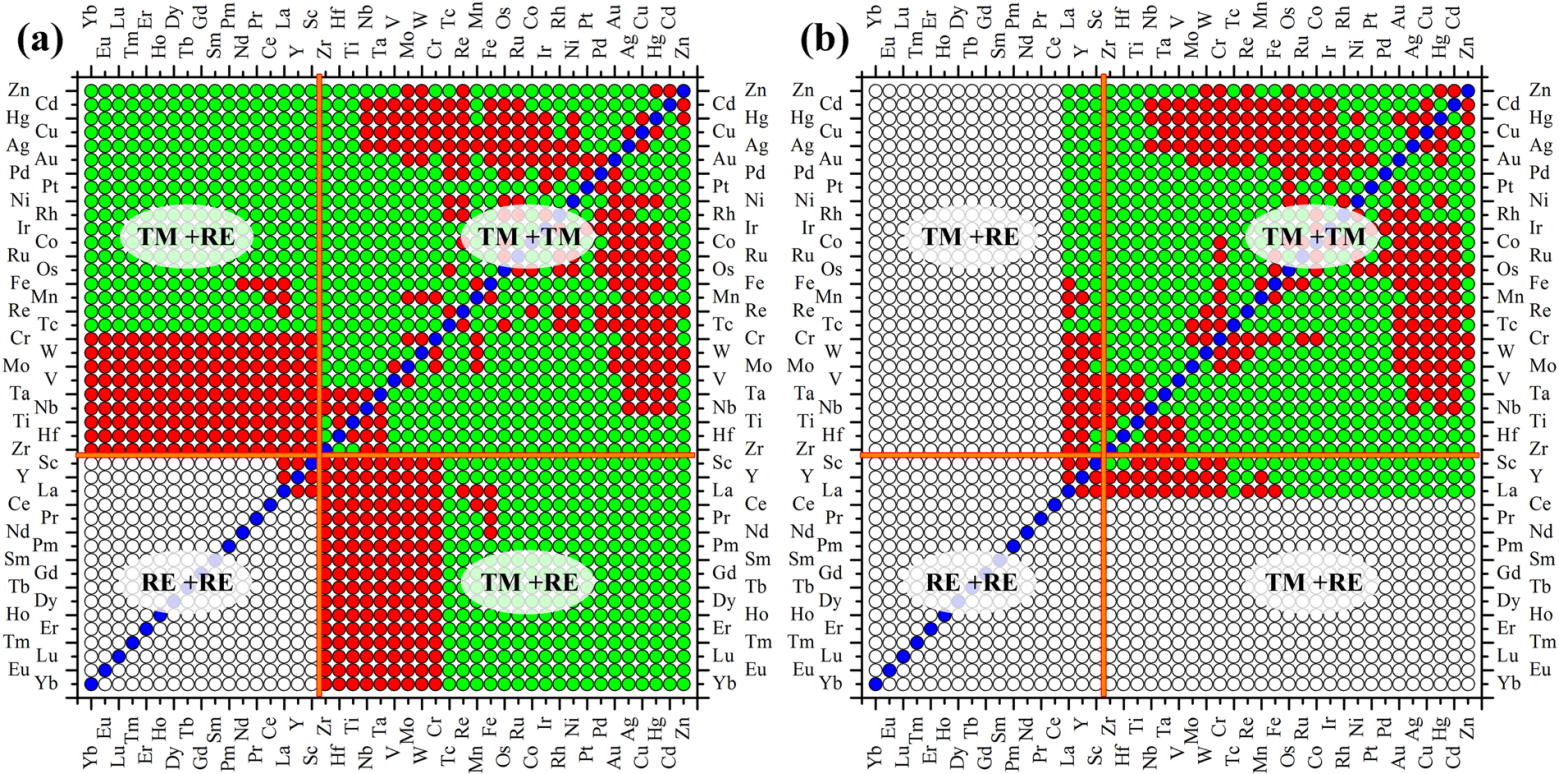
The miscibility map of binary alloy systems ordered by the slightly modified Pettifor chemical scale based on (**a**) Miedema’s theory, and (**b**) HTFP calculations. Three different regions are divided as: TM+TM indicates binary transition metal alloy systems, TM+RE for the combinations of transition metals and rare earth metals, RE+RE for the combinations of binary rare earth metals.

Miedema’s theory is generally regarded as the most successful empirical model to describe the energy effects during alloying. The electronegativity difference and electronic density discontinuity, used as parameters, can be regarded as a 2-dimensional (2D) basis for alloy behavior since it can separate the positive and negative systems with a reasonable precision. As shown in Fig. S2 (in the Supplemental Materials), however, the single parameter of Miedema’s model cannot separate unambiguously the immiscible systems into large clusters. However, assigning two coordinates to each transition element it was possible to separate all binary alloys with positive heats of formation from those with negative ones. The existence of intermetallic compounds in the binary phase diagram indicates a negative formation enthalpy. The same applies to the mutual solute solubility. A negative formation enthalpy is expected if one or more intermetallic compounds occur in the phase diagram, or if there is an appreciable solid solubility between the given elements. If neither condition is satisfied, a positive formation enthalpy is expected. Figure 4a shows the miscibility map of binary alloy systems ordered by the slightly modified Mendeleev number or the Pettifor chemical scale based on Miedema’s theory. Compared with Fig. 2b (or Fig. S3 in the Supplemental Materials), it is clear that the majority of the clustering region matches to each other with only minor disagreements, validating the success of Miedema’s theory. We recall the equation of Г parameter for formation enthalpy in the form^5, 14^:

1 $${\rm{\Gamma }}=\frac{1}{{({{\rm{n}}}_{{\rm{ws}}}^{-1/3})}_{{\rm{av}}}}\{-{\rm{P}}{({{\rm{\Delta }}{\rm{\Phi }}}^{\ast })}^{2}+{\rm{Q}}{({{\rm{\Delta }}n}_{{\rm{ws}}}^{1/3})}^{2}\}$$

Accordingly, the value of ${\rm{\Gamma }}=0$ should separate the positive (immiscible) and negative (miscible) systems. A zero value of Г means that

2 $$-{\rm{P}}{({{\rm{\Delta }}{\rm{\Phi }}}^{\ast })}^{2}+{\rm{Q}}{({{\rm{\Delta }}n}_{{\rm{ws}}}^{1/3})}^{2}=0$$

Thus, a straight line in a plot between ${{\rm{\Delta }}{\rm{\Phi }}}^{\ast }$ versus ${{\rm{\Delta }}n}_{{\rm{ws}}}^{1/3}$ should separate regions with positive and negative formation enthalpy.

**Figure 5:**
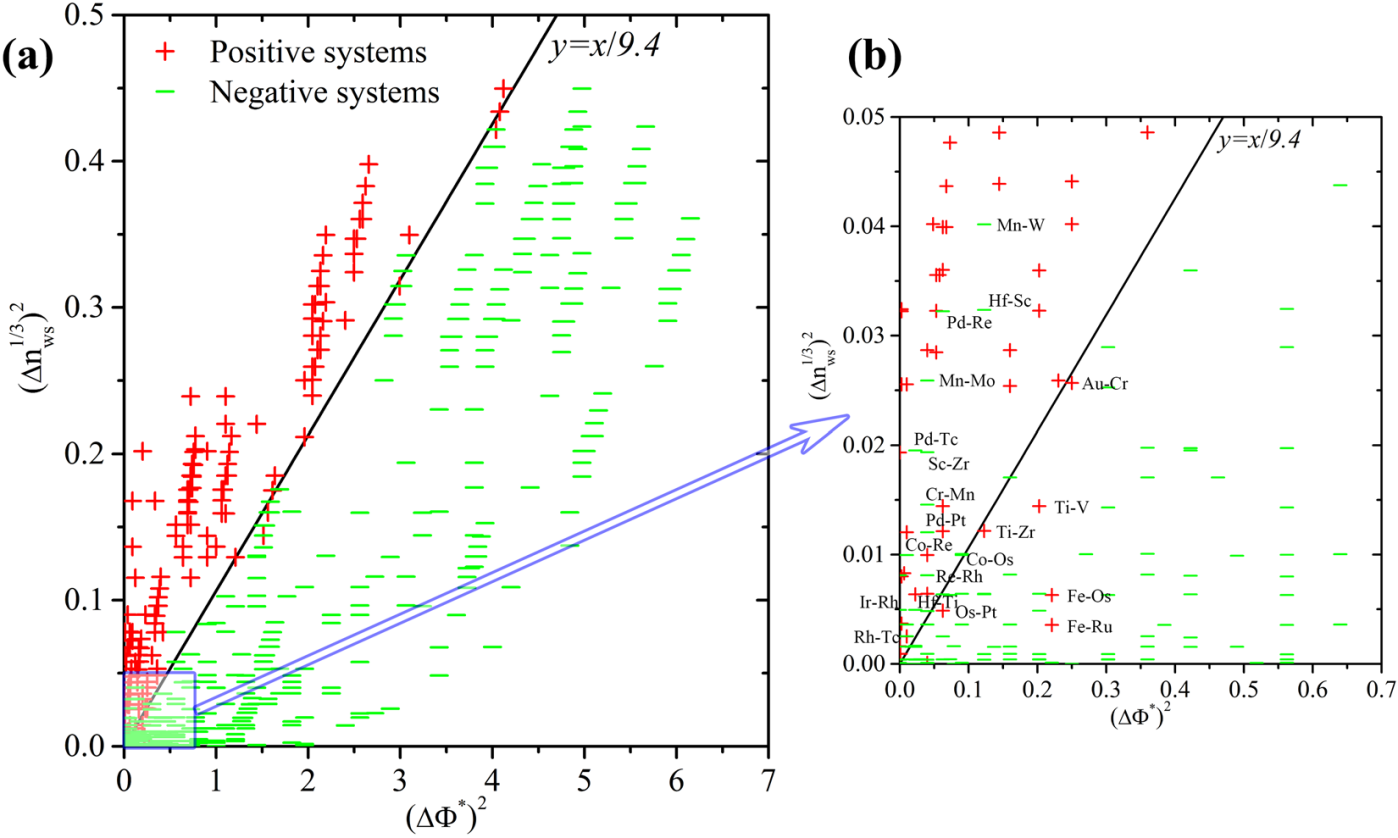
(**a**) The separation map of miscible and immiscible systems coordinated by the two Miedema’s parameters as two-dimensional axes. Plus symbols ‘+’ represents the immiscible systems, while minus symbols ‘−’ stands for the miscible systems with negative formation enthalpy, i.e. no intermetallic compounds exist or solubilities are generally smaller 10%. (**b**) The zoomed-in region with ${({{\rm{\Delta }}{\rm{\Phi }}}^{\ast })}^{2}$ from 0 to 0.7 and $({{\rm{\Delta }}n}_{{\rm{w}}{\rm{s}}}^{1/3}{)}^{2}$ from 0 to 0.05.

**Figure 6:**
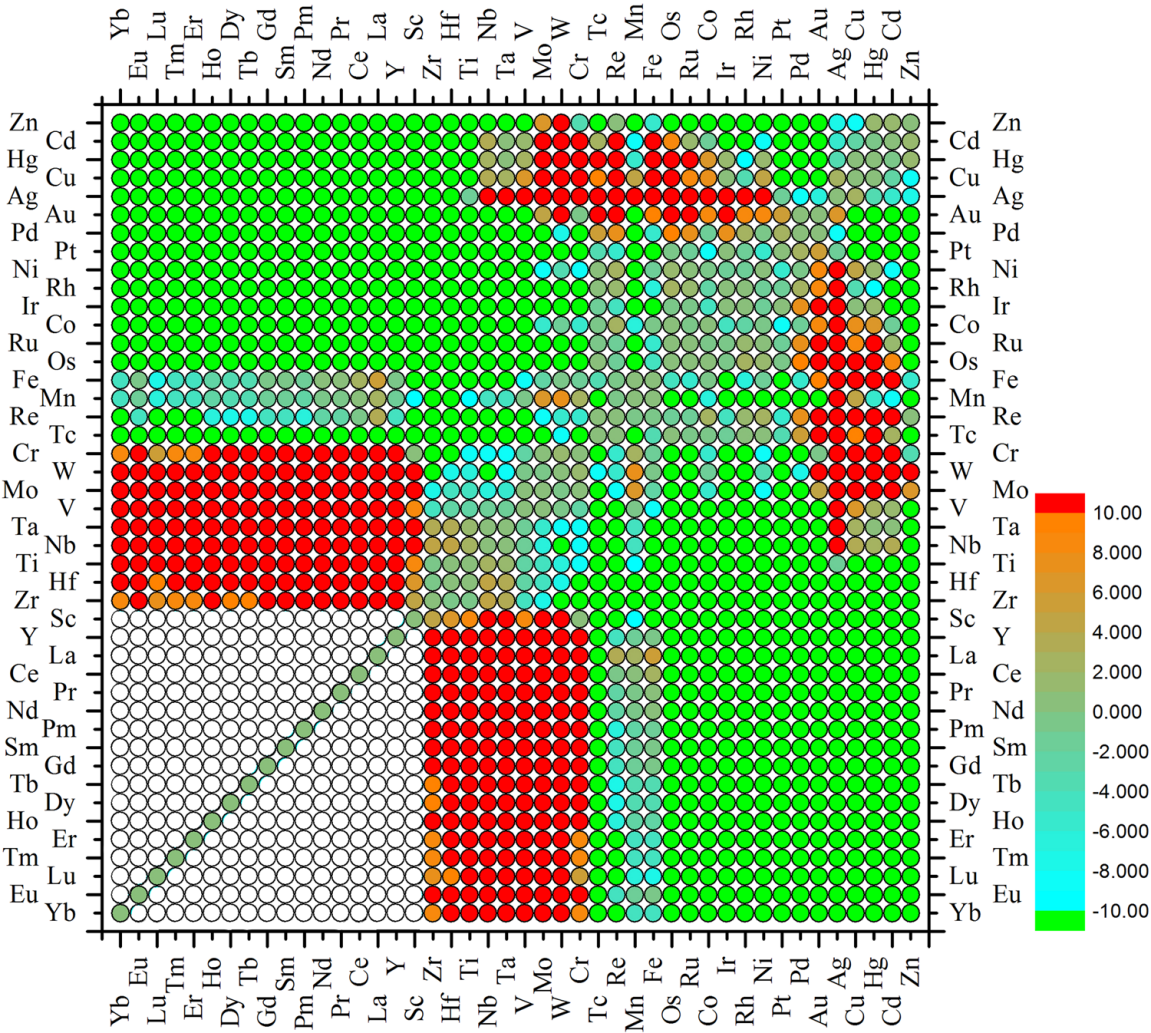
The distribution map of formation enthalpies of binary alloy systems with 1:1 stoichiometry calculated by Miedema’s model to indicate the distribution regions of highly miscible and immiscible systems. Red and green colored circles correspond to the positive formation enthalpy larger than 10kJ/mol and negative ones lower than −10kJ/mol respectively. The white symbols represent the systems with the unavailable experimental information and are not considered in the present study. The elemental sequence is ordered by the slightly modified Pettifor chemical scale.

Figure 5 shows such plot for a large number of TM-TM and TM-RE alloys. Indeed the straight line y = x/9.4 separates two regions: one for binary alloy systems with negative formation enthalpy (green minus symbols) and one for systems with positive formation enthalpy (red plus symbols). The straight line gives the empirical value of the ratio Q/P = 9.4. As seen in Fig. 5a, Miedema’s model provides a clear distinction between positive and negative systems when their formation enthalpy is sufficiently large. The agreement between the prediction and our data mining results is beyond 94%, which corresponds to large values of ${{\rm{\Delta }}{\rm{\Phi }}}^{\ast }$ or ${{\rm{\Delta }}n}_{{\rm{ws}}}^{1/3}$. However, when we zoom in the region with small values of ${({{\rm{\Delta }}{\rm{\Phi }}}^{\ast })}^{2}$ from 0 to 0.7 and $({{\rm{\Delta }}n}_{{\rm{w}}{\rm{s}}}^{1/3}{)}^{2}$ from 0 to 0.05, as seen in Fig. 5b, the separation between the “positive” and “negative” systems is not unambiguous. This illustrates the aforementioned weakness of Miedema’s model when it is used for binary alloy systems with formation enthalpies close to zero. A further demonstration of the correlation between the miscibility and formation enthalpies by Miedema’s theory is shown in Fig. 6. It shows clearly that the distributions of highly positive (>10 kJ/mol) and negative (<−10kJ/mol) binary alloy systems calculated by Miedema’s model correspond to the regions of highly miscible and immiscible systems classified in the present studies (see Fig. 2b).

Table 2 lists the 46 immiscible binary systems that exhibit disagreement between the results of our data mining-generated map and Miedema’s theory. It should be noted that our choice is based on the estimation on the reliability and validity of the available information and similarity of different elements. The inconsistency between our choice and Miedema’s theory is about 5%. In view of the uncertainty of each method, these systems warrant further careful study, both experimentally and theoretically.

**Table 2:** The binary alloy systems whose miscibility shows disagreement between present data mining and Miedema’s theory.

Systems	Alloying behavior		Systems	Alloying behavior	
	Present	Miedema(kJ/mol)		Present	Miedema(kJ/mol)
Au-Cr	+	−0.2	Ir-Rh	−	+0.8
Au-Os	−	+21.8	Mn-Mo	−	+6.0
Cd-Co	+	−1.8	Mn-W	−	+7.6
Cd-Ir	+	−12.6	Mn-Yb	+	−4.2
Cd-Mn	+	−8.1	Mo-V	−	+0.0
Ce-Fe	−	+2.5	Mo-Zn	−	+6.3
Ce-Re	+	−0.1	Nb-Ta	−	+0.0
Co-Os	−	+0.1	Ni-Re	−	+2.8
Co-Re	−	+2.5	Ni-Tc	−	+0.7
Cr-Mn	−	+2.6	Os-Pt	+	−0.6
Cu-Hg	−	+0.4	Os-Rh	−	+2.5
Cu-Ni	−	+4.3	Os-Ru	−	+0.1
Eu-Fe	+	−1.0	Os-Tc	−	+0.2
Eu-Mn	+	−1.6	Os-Zn	+	−13.7
Fe-Nd	−	+0.7	Pd-Pt	−	+2.4
Fe-Os	+	−4.9	Pd-Re	−	+7.9
Fe-Pr	−	+0.7	Pr-Re	+	−2.5
Fe-Ru	+	−5.6	Re-Rh	−	+1.2
Hf-Sc	−	+6.2	Re-Tc	−	+0.2
Hf-Ti	−	+0.2	Rh-Ru	−	+1.5
Hg-Ni	−	+1.1	Sc-Zr	−	+4.9
Hg-Zn	−	+1.2	Ti-Zr	+	−0.3
Tc-Ce	+	−25.9	Tc-Pr	+	−28.2

The present choice is based on the estimation on the reliability of the available information and elemental similarity. Plus symbol represents the immiscible systems, and minus symbol indicates the miscible or compound-formation systems.

For comparison, we have also constructed the miscibility map based on the HTFP data shown in Fig. 4b. This choice of the miscibility properties is based on the results of HTFP data by Curtarolo *et al*.^25, 32–34, 36–40^. One can see that there is some disagreement (~10%) with the results in Figs 2b and 4b. This implies that much more future work based on HTFP calculations is needed, and our present work shows the direction. For instance, further HTFP calculations based on a larger structure prototype library, e.g. ICSD or PCD, may help to validate the present results, although it is much computationally expensive for the TM-RE and RE-RE systems.

## Discussion

The data mining presented in this paper (and in the prior cited studies^4^) provides a unique approach to discover novel materials, their properties and the supporting physical mechanisms. Our present study demonstrates a simple and illustrative example of obtaining the miscibility map of binary alloy systems from a large amount of raw data sources. It also provides a general pathway for one-, two- and three-dimensional spaces. The basic idea of data mining is not limited to the binary alloys, and therefore one may extend it for multicomponent alloy systems. To be noted that Miedema’s theory is also applicable for ternary, quaternary and multicomponent systems via geometrical methods^20^, while the HTFP mothed can also be readily applied for the ternary and even higher-order alloys when their structures are known or can be predicted. The success of Miedema’s model and the HTFP method is clearly revealed, and some outliers are identified for further experimental and theoretical investigation. Because of the scarcity of reliable experimental and first-principles data for binary systems consisting of dissimilar lanthanides, it is of particular urgency to perform HTFP calculations on this group of systems.

## Methods

### Data collection and classification rule

A total of 946 combinations exist for binary alloy phase diagrams consisting of transition and lanthanide metals, of which about 800 are available in the Landolt-Boernstein Database^7^, in the “Desk handbook: Phase diagrams for binary alloys” compiled by Okamoto^9^, and in the ASM alloy phase diagrams database organized by the ASM community^8^. In addition, the experimental standard formation enthalpies determined by Kleppa *et al*.^24–35^ provide a further validation of the compound-formation systems. The recent computational binary alloy project, hosted in the *aflowlib.org* consortium repository^40^, contains the formation enthalpies for hundreds of thousands of intermetallics comprising all of the transition metal systems. However, this repository does not include any alloying data between transition and lanthanide metals, and therefore a throughout data mining is much demanded for further design of novel alloys.

The binary solid phases consisting of elements A and B, may be classified into three groups: mechanical mixtures, solid solutions, and ordered intermetallic compounds. The mechanical mixture corresponds to binary systems with a positive formation enthalpy, while the intermetallic compound is favored for a negative formation enthalpy. The solid solution has three subtypes: clustered solid solution, disordered solid solution, or ordered solid solution. For the former two cases, the different kinds of atoms are energetically preferable to mix, while for the last case the different kinds of atoms prefer to segregate into different clusters. In comparison to the formation of intermetallic compounds with a negative formation enthalpy, the first two cases may be classified as poorly miscible systems, while the latter case can be classified as poorly immiscible systems since the formation enthalpy is close to zero^20^.

**Figure 7:**
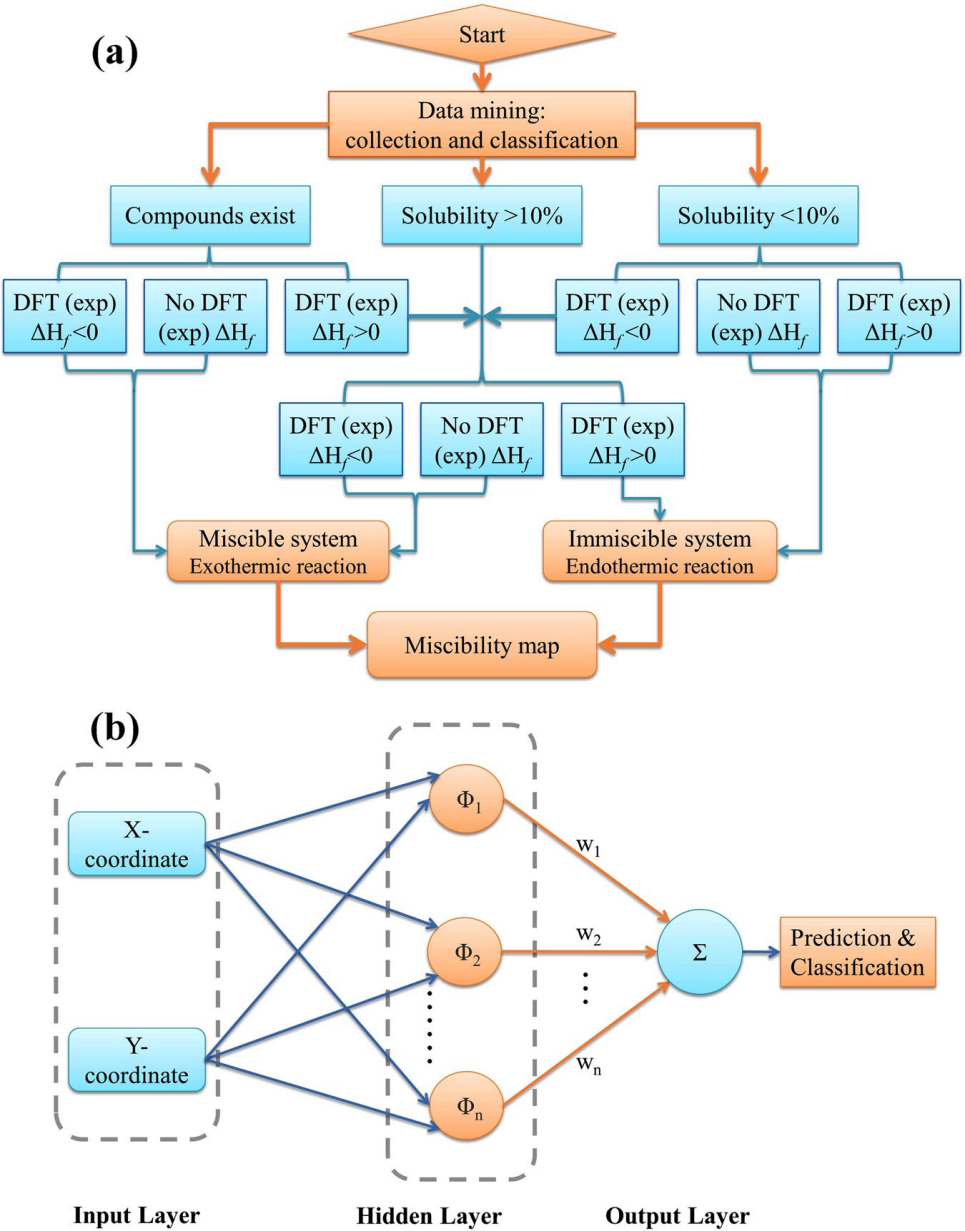
(**a**) A classification flowchart used in data mining to distinguish the miscible and immiscible systems based on the binary alloy phase diagrams, thermodynamic data and high-throughput first principles (HTFP) calculations. (**b**) A schematic diagram of radial basis function artificial neural network (RBF-ANN) for the prediction of the unknown systems.

With this classification rule of different solid phases and the five alloy databases at hand, a data mining scheme is proposed to separate the immiscible systems from those miscible ones in which the homogeneous disordered solid solution and intermetallic compounds may form. Figure 7a shows the proposed flowchart of the classification scheme that we shall use. Note that the miscible systems are those in which one or more intermetallic compounds, or one or more ordered solid phases, or a disordered solid solution exist with negative formation enthalpy. The immiscible systems correspond to those with positive formation enthalpy, in which case either a mechanical mixture or clustered solid solution may form at low temperature.

### Artificial neural network algorithm for prediction

We use a radial basis function artificial neural network (RBF-ANN) algorithm to predict the immiscibility of an unknown system. The RBF-ANN has a three-layer structure: an input layer, a hidden layer and a linear output layer, as shown in Fig. 7b. In this work, the input and output of RBF-ANN are the map coordinates e.g. the “X- and Y-coordinate” in Fig. 7b and the miscible/immiscible classification, respectively. The input layer only transfers input data to the hidden layer. The hidden layer uses Gaussian functions as the radial basis function *Ф*. It can produce a localized response to the input and introduce non-linearity into the network^50^.

3 $${{\rm{\Phi }}}_{i}(X)=\exp [-\frac{{\Vert X-{c}_{i}\Vert }^{2}}{2{\sigma }_{i}^{2}}],\,i=1,\,2,\,\ldots ,\,{\rm{n}}$$

Where X is the input vector, c_i_ is the centroid of the basis function of the i^th^ hidden layer node, and σ_i_ is the variance of basis function.

Before the RBF-ANN is trained, all data are scaled to fit within the interval [−1,1]. During the training processes, the center vectors c_i_ and variances σ_i_ of basis functions in the hidden layer are determined using k-means clustering^51^. First the k samples are randomly chosen as the initial values of the center vectors c_i_, and the other samples are classified to form k subclasses according to the Euclidean distance between samples and center vectors. The new center vectors (i.e. the mean values of subclass data) are continually calculated by an iterative technique until they do not change. After the center vectors are determined, the variances σ_i_ can be obtained by the maximum distance among the center vectors. Subsequently, the weights w_i_ between the hidden and output layers (see Fig. 7b) are modified by the least mean square algorithm. The training of the network is completed after the number of neurons in the hidden layer reaches the sample dataset size (n = 770). The mean square errors of training are 4.7 × 10^−2^. We then use this network topological structure to predict the classification of unknown alloy systems.

## Electronic supplementary material

Supplementary Information
