# Supplementary material for: An informatics guided classification of miscible and immiscible binary alloy systems
Source: Sci Rep. 2017 Aug 29;7:9577. doi: 10.1038/s41598-017-09704-1 (PMC5575349; doi:10.1038/s41598-017-09704-1)
Supplement: Supplementary file 1 — Supplementary Information [file 41598_2017_9704_MOESM1_ESM.doc]

**An informatics guided classification of miscible and immiscible binary alloy systems**

R. F. Zhang^1,*^, X. F. Kong^1^, H. T. Wang^2^, S. H. Zhang^1^, D. Legut^3^, S. H. Sheng^1^, S. Srinivasan^4^, K. Rajan^5^, and T. C. Germann^6^

^1^School of Materials Science and Engineering, and International Research Institute for Multidisciplinary Science, Beihang University, Beijing 100191, P. R. China

^2^CAS Key Laboratory of Nuclear Materials and Safety Assessment, Institute of Metal Research, Chinese Academy of Sciences, Shenyang 110016, P.R. China

^3^IT4Innovations Center & Nanotechnology Centre, VSB-Technical University of Ostrava, CZ-70833 Ostrava, Czech Republic

^4^Plant Sciences Institute, Iowa State University, 2031 Roy J. Carver Co-Lab, Ames, IA 50011, USA

^5^Department of Materials Design and Innovation, University at Buffalo-State University of New York, 311 Bell Hall, Buffalo, NY 14260, USA

^6^Theoretical Division, Los Alamos National Laboratory, Los Alamos, NM, 87545, USA

Correspondence and requests for materials should be addressed to R. F. Z. (e-mail: [zrf@buaa.edu.cn](mailto:zrf@buaa.edu.cn))

**Table S1.** The adopted values of Pauling electronegativity (Pen), Teatum metallic radii of elements with coordinate number CN12 (Rij), Martynov-Batsanov electronegativity (Men), Zunger’s pseudopotential radii sum (Rsp), Miedema’s work function (Phi), Miedema’s electron density at the Wigner-Seitz cell boundary (Nws), Miedema’s molar volume (Vm), and Mendeleev number or Pettifor chemical scale (Pcs), for the ordering of 44 elements including 27 pseudo-transition metals and 17 pseudo-lanthanides elements.

| Elements | Group 1 | Group 2 | | Group 3 | | Group 4 | | | Group 5 |
| --- | --- | --- | --- | --- | --- | --- | --- | --- | --- |
|  | AN | Pen | Rij | Men | Rsp | Phi | Nws | Vm | Pcs |
| Ag | 47 | 1.93 | 1.445 | 1.07 | 2.375 | 4.35 | 2.52 | 10.25 | 1.07 |
| Au | 79 | 2.54 | 1.442 | 1.19 | 2.66 | 5.15 | 3.87 | 10.2 | 1.19 |
| Cd | 48 | 1.69 | 1.568 | 1.4 | 2.215 | 4.05 | 1.91 | 13 | 1.4 |
| Ce | 58 | 1.12 | 1.846 | 1.1 | 4.5 | 3.18 | 1.69 | 21.62 | 1.1 |
| Co | 27 | 1.88 | 1.252 | 1.72 | 2.02 | 5.1 | 5.36 | 6.7 | 1.72 |
| Cr | 24 | 1.66 | 1.282 | 2 | 2.44 | 4.65 | 5.18 | 7.23 | 2 |
| Cu | 29 | 1.9 | 1.278 | 1.08 | 2.04 | 4.45 | 3.18 | 7.12 | 1.08 |
| Dy | 66 | 1.22 | 1.775 | 1.15 | 3.67 | 3.21 | 1.82 | 19 | 1.15 |
| Er | 68 | 1.24 | 1.758 | 1.2 | 3.63 | 3.22 | 1.86 | 18.45 | 1.2 |
| Eu | 63 | 1.2 | 1.798 | 1.15 | 3.94 | 3.2 | 1.77 | 19.97 | 1.15 |
| Fe | 26 | 1.83 | 1.274 | 1.67 | 2.11 | 4.93 | 5.55 | 7.09 | 1.67 |
| Gd | 64 | 1.2 | 1.801 | 1.1 | 3.91 | 3.2 | 1.77 | 19.9 | 1.1 |
| Hf | 72 | 1.3 | 1.58 | 1.73 | 2.91 | 3.6 | 3.05 | 13.45 | 1.73 |
| Hg | 80 | 2 | 1.594 | 1.49 | 2.41 | 4.2 | 1.91 | 14.08 | 1.49 |
| Ho | 67 | 1.23 | 1.767 | 1.2 | 3.65 | 3.22 | 1.82 | 18.76 | 1.2 |
| Ir | 77 | 2.2 | 1.357 | 1.87 | 2.628 | 5.55 | 6.13 | 8.52 | 1.87 |
| La | 57 | 1.1 | 1.877 | 1.35 | 3.08 | 3.17 | 1.64 | 22.55 | 1.35 |
| Lu | 71 | 1.27 | 1.735 | 1.2 | 3.37 | 3.22 | 1.91 | 17.77 | 1.2 |
| Mn | 25 | 1.55 | 1.254 | 2.04 | 2.22 | 4.45 | 4.17 | 7.35 | 2.04 |
| Mo | 42 | 2.16 | 1.4 | 1.94 | 2.72 | 4.65 | 5.55 | 9.4 | 1.94 |
| Nb | 41 | 1.6 | 1.468 | 2.03 | 2.76 | 4.05 | 4.41 | 10.8 | 2.03 |
| Nd | 60 | 1.14 | 1.822 | 1.2 | 3.99 | 3.19 | 1.73 | 20.58 | 1.2 |
| Ni | 28 | 1.91 | 1.246 | 1.76 | 2.18 | 5.2 | 5.36 | 6.6 | 1.76 |
| Os | 76 | 2.2 | 1.353 | 1.85 | 2.65 | 5.4 | 6.33 | 8.45 | 1.85 |
| Pd | 46 | 2.2 | 1.376 | 2.08 | 2.45 | 5.45 | 4.66 | 8.9 | 2.08 |
| Pm | 61 | 1.13 | 1.809 | 1.15 | 3.99 | 3.19 | 1.77 | 20.25 | 1.15 |
| Pr | 59 | 1.13 | 1.828 | 1.1 | 4.48 | 3.19 | 1.73 | 20.79 | 1.1 |
| Pt | 78 | 2.28 | 1.387 | 1.91 | 2.7 | 5.65 | 5.64 | 9.1 | 1.91 |
| Re | 75 | 1.9 | 1.375 | 2.06 | 2.68 | 5.2 | 6.33 | 8.85 | 2.06 |
| Rh | 45 | 2.28 | 1.345 | 1.99 | 2.52 | 5.4 | 5.45 | 8.3 | 1.99 |
| Ru | 44 | 2.2 | 1.339 | 1.97 | 2.605 | 5.4 | 6.13 | 8.2 | 1.97 |
| Sc | 21 | 1.36 | 1.641 | 1.5 | 2.75 | 3.25 | 2.05 | 15.03 | 1.5 |
| Sm | 62 | 1.17 | 1.802 | 1.2 | 4.14 | 3.2 | 1.77 | 20.01 | 1.2 |
| Ta | 73 | 1.5 | 1.467 | 1.94 | 2.79 | 4.05 | 4.33 | 10.81 | 1.94 |
| Tb | 65 | 1.2 | 1.783 | 1.2 | 3.89 | 3.21 | 1.82 | 19.32 | 1.2 |
| Tc | 43 | 1.9 | 1.365 | 2.18 | 2.65 | 5.3 | 5.93 | 8.64 | 2.18 |
| Ti | 22 | 1.54 | 1.462 | 1.86 | 2.58 | 3.8 | 3.51 | 10.58 | 1.86 |
| Tm | 69 | 1.25 | 1.747 | 1.2 | 3.6 | 3.22 | 1.86 | 18.12 | 1.2 |
| V | 23 | 1.63 | 1.346 | 2.22 | 2.43 | 4.25 | 4.41 | 8.36 | 2.22 |
| W | 74 | 2.36 | 1.408 | 1.79 | 2.735 | 4.8 | 5.93 | 9.55 | 1.79 |
| Y | 39 | 1.22 | 1.773 | 1.41 | 2.94 | 3.2 | 1.77 | 19.9 | 1.41 |
| Yb | 70 | 1.1 | 1.741 | 1.1 | 3.59 | 3.22 | 1.86 | 17.97 | 1.1 |
| Zn | 30 | 1.65 | 1.394 | 1.44 | 1.88 | 4.1 | 2.3 | 9.17 | 1.44 |
| Zr | 40 | 1.33 | 1.602 | 1.7 | 2.825 | 3.45 | 2.8 | 14 | 1.7 |

**Table S2.** Total 262 immiscible binary systems separated from 813 binary alloy systems through the present data mining technique. Significant feature is found that, Cr, Nb, Ta, W, Mo -based systems possess the largest number of immiscibility with other elements.

| Group | Number | Binary alloy systems |
| --- | --- | --- |
| Ag-based | 17 | Ag-Co, Ag-Cr, Ag-Cu, Ag-Fe, Ag-Ir, Ag-Mn, Ag-Mo, Ag-Nb, Ag-Ni, Ag-Os, Ag-Re, Ag-Rh, Ag-Ru, Ag-Ta, Ag-Tc, Ag-V, Ag-W |
| Au-based | 13 | Au-Co, Au-Cr, Au-Fe, Au-Ir, Au-Mo, Au-Ni, Au-Os, Au-Pt, Au-Re, Au-Rh, Au-Ru, Au-Tc, Au-W |
| Cd-based | 15 | Cd-Co, Cd-Cr, Cd-Fe, Cd-Ir, Cd-Mn, Cd-Mo, Cd-Nb, Cd-Os, Cd-Re, Cd-Ru, Cd-Ta, Cd-Tc, Cd-V, Cd-W, Cd-Zn |
| Ce-based | 12 | Ce-Cr, Ce-Hf, Ce-Mn, Ce-Mo, Ce-Nb, Ce-Re, Ce-Ta, Ce-Ti, Ce-Tc, Ce-V, Ce-W, Ce-Zr |
| Co-based | 5 | Co-Cu, Co-Hg, Ag-Co, Au-Co, Cd-Co |
| Cr-based | 24 | Cr-Cu, Cr-Dy, Cr-Er, Cr-Eu, Cr-Gd, Cr-Hg, Cr-Ho, Cr-La, Cr-Lu, Cr-Mo, Cr-Nd, Cr-Pm, Cr-Pr, Cr-Sc, Cr-Sm, Cr-Tb, Cr-Tm, Cr-W, Cr-Y, Cr-Yb, Ag-Cr, Au-Cr, Cd-Cr, Ce-Cr |
| Cu-based | 15 | Cu-Fe, Cu-Ir, Cu-Mn, Cu-Mo, Cu-Nb, Cu-Os, Cu-Re, Cu-Ru, Cu-Ta, Cu-Tc, Cu-V, Cu-W, Ag-Cu, Co-Cu, Cr-Cu |
| Dy-based | 9 | Dy-Hf, Dy-Mo, Dy-Nb, Dy-Ta, Dy-Ti, Dy-V, Dy-W, Dy-Zr, Cr-Dy |
| Er-based | 9 | Er-Hf, Er-Mo, Er-Nb, Er-Ta, Er-Ti, Er-V, Er-W, Er-Zr, Cr-Er |
| Eu-based | 11 | Eu-Fe, Eu-Hf, Eu-Mn, Eu-Mo, Eu-Nb, Eu-Ta, Eu-Ti, Eu-V, Eu-W, Eu-Zr, Cr-Eu |
| Fe-based | 10 | Fe-Hg, Fe-La, Fe-Mn, Fe-Os, Fe-Ru, Ag-Fe, Au-Fe, Cd-Fe, Cu-Fe, Eu-Fe |
| Gd-based | 9 | Gd-Hf, Gd-Mo, Gd-Nb, Gd-Ta, Gd-Ti, Gd-V, Gd-W, Gd-Zr, Cr-Gd |
| Hf-based | 18 | Hf-Ho, Hf-La, Hf-Lu, Hf-Nb, Hf-Nd, Hf-Pm, Hf-Pr, Hf-Sm, Hf-Ta, Hf-Tb, Hf-Tm, Hf-Y, Hf-Yb, Ce-Hf, Dy-Hf, Er-Hf, Eu-Hf, Gd-Hf |
| Hg-based | 13 | Hg-Ir, Hg-Mo, Hg-Nb, Hg-Os, Hg-Re, Hg-Ru, Hg-Ta, Hg-Tc, Hg-V, Hg-W, Co-Hg, Cr-Hg, Fe-Hg |
| Ho-based | 9 | Ho-Mo, Ho-Nb, Ho-Ta, Ho-Ti, Ho-V, Ho-W, Ho-Zr, Cr-Ho, Hf-Ho |
| Ir-based | 7 | Ir-Pd, Ir-Pt, Ag-Ir, Au-Ir, Cd-Ir, Cu-Ir, Hg-Ir |
| La-based | 14 | La-Mn, La-Mo, La-Nb, La-Re, La-Sc, La-Ta, La-Ti, La-V, La-W, La-Y, La-Zr, Cr-La, Fe-La, Hf-La |
| Lu-based | 9 | Lu-Mo, Lu-Nb, Lu-Ta, Lu-Ti, Lu-V, Lu-W, Lu-Zr, Cr-Lu, Hf-Lu |
| Mn-based | 8 | Mn-Yb, Ag-Mn, Cd-Mn, Ce-Mn, Cu-Mn, Eu-Mn, Fe-Mn, La-Mn |
| Mo-based | 23 | Mo-Nd, Mo-Pm, Mo-Pr, Mo-Sc, Mo-Sm, Mo-Tb, Mo-Tm, Mo-Y, Mo-Yb, Ag-Mo, Au-Mo, Cd-Mo, Ce-Mo, Cr-Mo, Cu-Mo, Dy-Mo, Er-Mo, Eu-Mo, Gd-Mo, Hg-Mo, Ho-Mo, La-Mo, Lu-Mo |
| Nb-based | 24 | Nb-Nd, Nb-Pm, Nb-Pr, Nb-Sc, Nb-Sm, Nb-Tb, Nb-Ti, Nb-Tm, Nb-Y, Nb-Yb, Nb-Zr, Ag-Nb, Cd-Nb, Ce-Nb, Cu-Nb, Dy-Nb, Er-Nb, Eu-Nb, Gd-Nb, Hf-Nb, Hg-Nb, Ho-Nb, La-Nb, Lu-Nb |
| Nd-based | 9 | Nd-Ta, Nd-Ti, Nd-V, Nd-W, Nd-Zr, Cr-Nd, Hf-Nd, Mo-Nd, Nb-Nd |
| Ni-based | 5 | Ni-Os, Ni-Rh, Ni-Ru, Ag-Ni, Au-Ni |
| Os-based | 10 | Os-Pd, Os-Pt, Os-Zn, Ag-Os, Au-Os, Cd-Os, Cu-Os, Fe-Os, Hg-Os, Ni-Os |
| Pd-based | 4 | Pd-Rh, Pd-Ru, Ir-Pd, Os-Pd |
| Pm-based | 9 | Pm-Ta, Pm-Ti, Pm-V, Pm-W, Pm-Zr, Cr-Pm, Hf-Pm, Mo-Pm, Nb-Pm |
| Pr-based | 11 | Pr-Re, Pr-Ta, Pr-Tc, Pr-Ti, Pr-V, Pr-W, Pr-Zr, Cr-Pr, Hf-Pr, Mo-Pr, Nb-Pr |
| Pt-based | 3 | Au-Pt, Ir-Pt, Os-Pt |
| Re-based | 9 | Re-Zn, Ag-Re, Au-Re, Cd-Re, Ce-Re, Cu-Re, Hg-Re, La-Re, Pr-Re |
| Rh-based | 4 | Ag-Rh, Au-Rh, Ni-Rh, Pd-Rh |
| Ru-based | 8 | Ag-Ru, Au-Ru, Cd-Ru, Cu-Ru, Fe-Ru, Hg-Ru, Ni-Ru, Pd-Ru |
| Sc-based | 9 | Sc-Ta, Sc-Ti, Sc-V, Sc-W, Sc-Y, Cr-Sc, La-Sc, Mo-Sc, Nb-Sc |
| Sm-based | 9 | Sm-Ta, Sm-Ti, Sm-V, Sm-W, Sm-Zr, Cr-Sm, Hf-Sm, Mo-Sm, Nb-Sm |
| Ta-based | 24 | Ta-Tb, Ta-Ti, Ta-Tm, Ta-Y, Ta-Yb, Ta-Zr, Ag-Ta, Cd-Ta, Ce-Ta, Cu-Ta, Dy-Ta, Er-Ta, Eu-Ta, Gd-Ta, Hf-Ta, Hg-Ta, Ho-Ta, La-Ta, Lu-Ta, Nd-Ta, Pm-Ta, Pr-Ta, Sc-Ta, Sm-Ta |
| Tb-based | 9 | Tb-Ti, Tb-V, Tb-W, Tb-Zr, Cr-Tb, Hf-Tb, Mo-Tb, Nb-Tb, Ta-Tb |
| Tc-based | 7 | Ag-Tc, Au-Tc, Cd-Tc, Cu-Tc, Hg-Tc, Ce-Tc, Pr-Tc |
| Ti-based | 21 | Ti-Tm, Ti-V, Ti-Y, Ti-Yb, Ti-Zr, Ce-Ti, Dy-Ti, Er-Ti, Eu-Ti, Gd-Ti, Ho-Ti, La-Ti, Lu-Ti, Nb-Ti, Nd-Ti, Pm-Ti, Pr-Ti, Sc-Ti, Sm-Ti, Ta-Ti, Tb-Ti |
| Tm-based | 9 | Tm-V, Tm-W, Tm-Zr, Cr-Tm, Hf-Tm, Mo-Tm, Nb-Tm, Ta-Tm, Ti-Tm |
| V-based | 22 | V-Y, V-Yb, Ag-V, Cd-V, Ce-V, Cu-V, Dy-V, Er-V, Eu-V, Gd-V, Hg-V, Ho-V, La-V, Lu-V, Nd-V, Pm-V, Pr-V, Sc-V, Sm-V, Tb-V, Ti-V, Tm-V |
| W-based | 24 | W-Y, W-Yb, W-Zn, Ag-W, Au-W, Cd-W, Ce-W, Cr-W, Cu-W, Dy-W, Er-W, Eu-W, Gd-W, Hg-W, Ho-W, La-W, Lu-W, Nd-W, Pm-W, Pr-W, Sc-W, Sm-W, Tb-W, Tm-W |
| Y-based | 11 | Y-Zr, Cr-Y, Hf-Y, La-Y, Mo-Y, Nb-Y, Sc-Y, Ta-Y, Ti-Y, V-Y, W-Y |
| Yb-based | 10 | Yb-Zr, Cr-Yb, Hf-Yb, Mn-Yb, Mo-Yb, Nb-Yb, Ta-Yb, Ti-Yb, V-Yb, W-Yb |
| Zn-based | 4 | Cd-Zn, Os-Zn, Re-Zn, W-Zn |
| Zr-based | 19 | Ce-Zr, Dy-Zr, Er-Zr, Eu-Zr, Gd-Zr, Ho-Zr, La-Zr, Lu-Zr, Nb-Zr, Nd-Zr, Pm-Zr, Pr-Zr, Sm-Zr, Ta-Zr, Tb-Zr, Tm-Zr, Ti-Zr, Y-Zr, Yb-Zr |


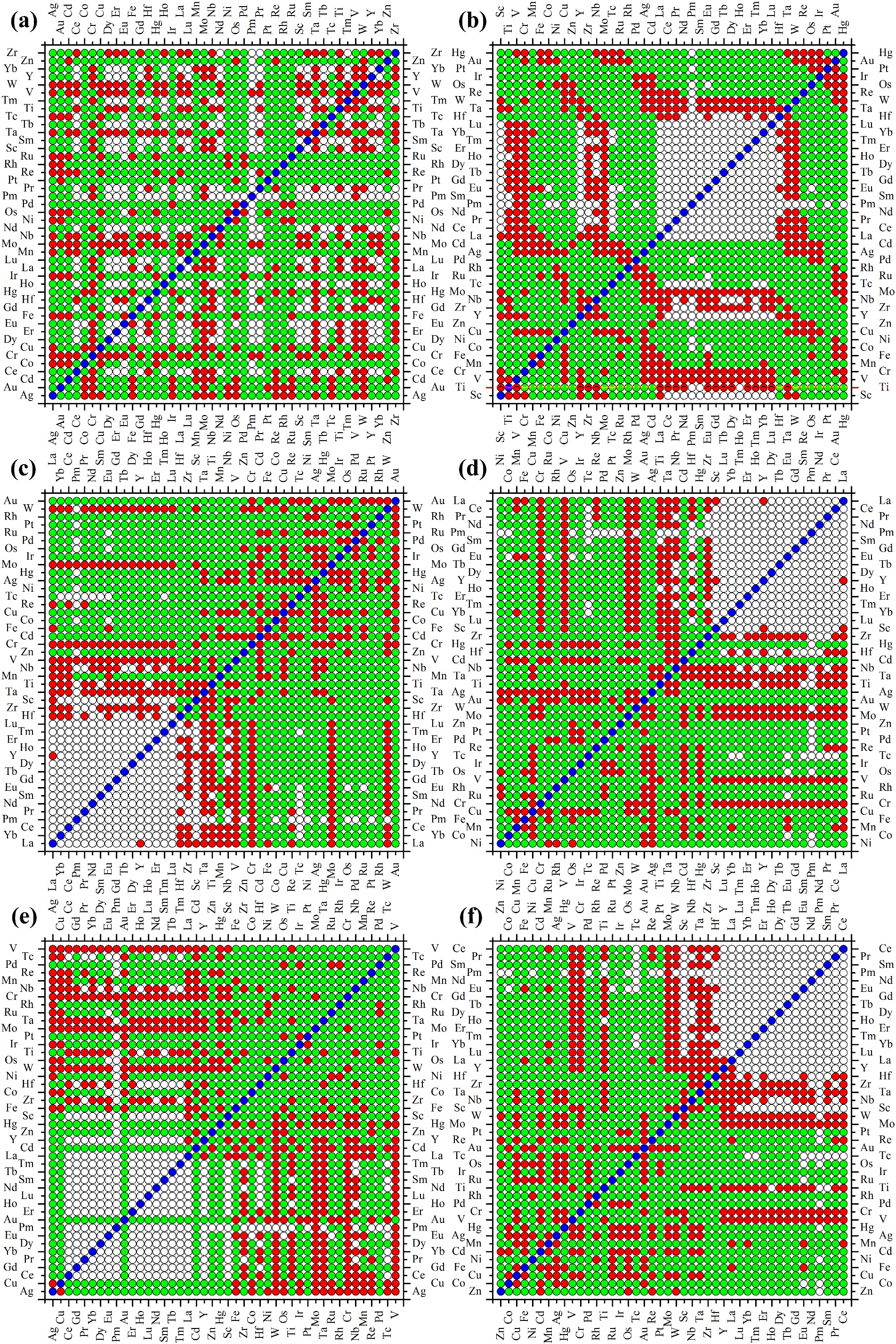


**Figure S1.** The clustering analysis of binary alloy systems, ordered by (a) Alphabet of elemental symbol, (b) Atomic number, (c) Pauling electronegtivity, (d) Teatum metallic atomic radii, (e) Martynov-Batsanov electronegativity, (f) Zunger’s pseudo radii sum. The red symbols indicate immiscible systems, and green symbols denote the miscible systems respectively.


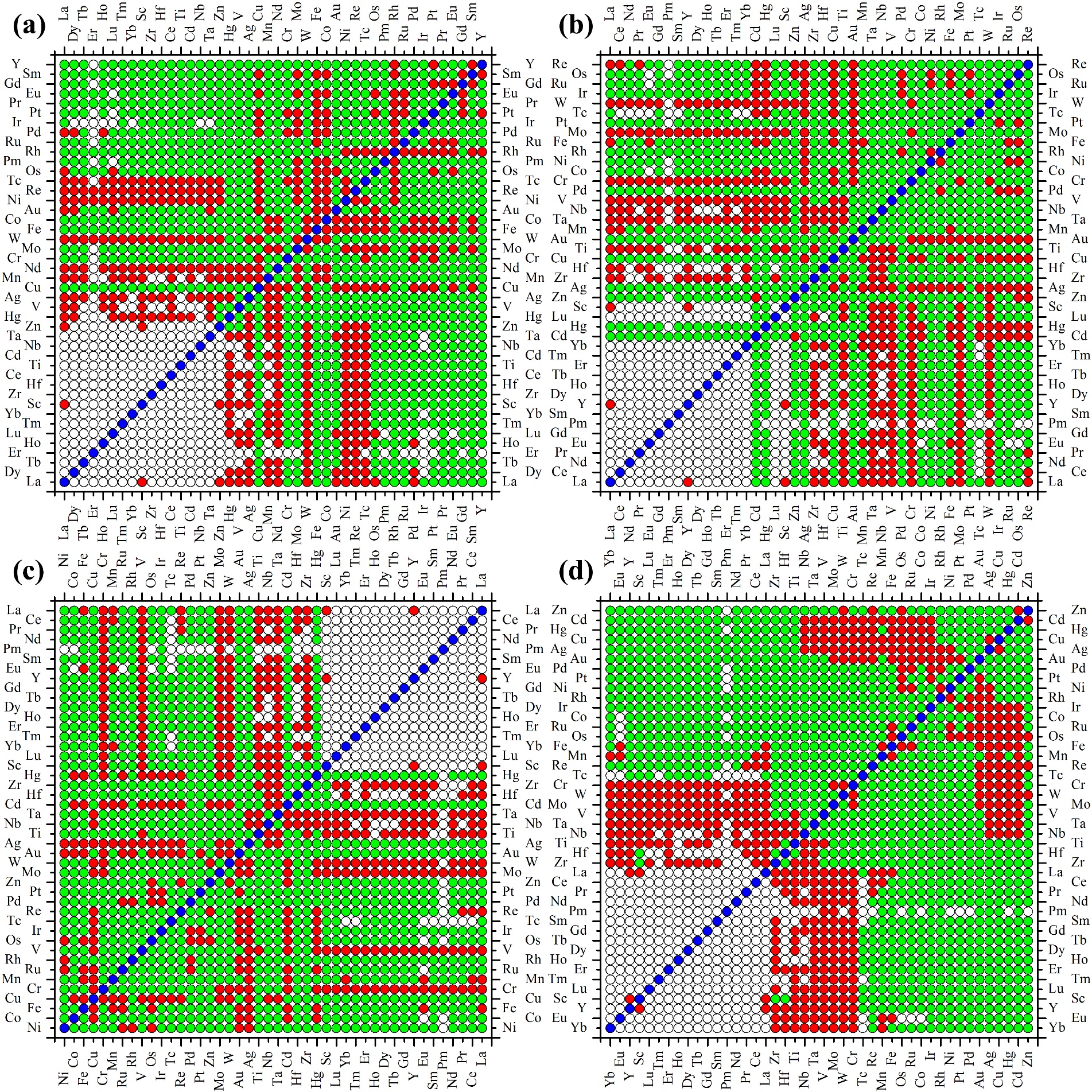


**Figure S2.** The cluster analysis of binary alloy systems, ordered by (a) Miedema’s electronegativity, (b) Miedema’s electron density, (c) Miedema’s molar volume, (d) Pettifor chemical scale (or Mendeleev number). The red symbols indicate immiscible systems, and green symbols denote the miscible systems respectively.
